# Text mining for improved exposure assessment

**DOI:** 10.1371/journal.pone.0173132

**Published:** 2017-03-03

**Authors:** Kristin Larsson, Simon Baker, Ilona Silins, Yufan Guo, Ulla Stenius, Anna Korhonen, Marika Berglund

**Affiliations:** 1 Institute of Environmental Medicine, Karolinska Institute, Stockholm, Sweden; 2 Computer Laboratory, University of Cambridge, Cambridge, United Kingdom; 3 Language Technology Lab, DTAL, University of Cambridge, Cambridge, United Kingdom; Garvan Institute of Medical Research, AUSTRALIA

## Abstract

Chemical exposure assessments are based on information collected via different methods, such as biomonitoring, personal monitoring, environmental monitoring and questionnaires. The vast amount of chemical-specific exposure information available from web-based databases, such as PubMed, is undoubtedly a great asset to the scientific community. However, manual retrieval of relevant published information is an extremely time consuming task and overviewing the data is nearly impossible. Here, we present the development of an automatic classifier for chemical exposure information. First, nearly 3700 abstracts were manually annotated by an expert in exposure sciences according to a taxonomy exclusively created for exposure information. Natural Language Processing (NLP) techniques were used to extract semantic and syntactic features relevant to chemical exposure text. Using these features, we trained a supervised machine learning algorithm to automatically classify PubMed abstracts according to the exposure taxonomy. The resulting classifier demonstrates good performance in the intrinsic evaluation. We also show that the classifier improves information retrieval of chemical exposure data compared to keyword-based PubMed searches. Case studies demonstrate that the classifier can be used to assist researchers by facilitating information retrieval and classification, enabling data gap recognition and overviewing available scientific literature using chemical-specific publication profiles. Finally, we identify challenges to be addressed in future development of the system.

## 1. Introduction

**Fig 1:**
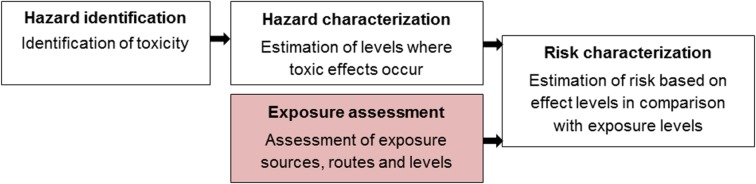
Chemical risk assessment. The process of a chemical risk assessment includes exposure assessment, hazard identification, hazard characterization and risk characterization [1, 2].

Humans are constantly exposed to a large number of chemicals present in food, water, air, dust, soil and consumer products via ingestion, inhalation and dermal absorption. Many of these chemicals have known or suspected toxic effects that can cause disorders and diseases at certain exposure levels. Aiming to estimate whether a population may be at risk at the current levels of exposure, risk assessments of chemicals are performed (Fig 1). One cornerstone in the risk assessment process is the exposure assessment in which the magnitude, frequency and duration of exposure are estimated or measured (WHO/IPCS 2004).

Exposure assessment methods include both indirect methods, such as exposure modeling and exposure calculations based on environmental measurements and questionnaire data, and direct measurements, such as human biomonitoring (HBM) and personal monitoring. HBM is the measurement of exposure biomarkers (chemicals or chemical metabolites) and effect biomarkers (indicators of effects caused by chemical exposure) in human body tissues or fluids, such as blood, hair and urine [3].

To assess the total exposure to a chemical and evaluate the importance of different exposure routes, all available data should be considered. In recent years, published biomedical literature available from web-based databases (such as PubMed) has grown with double-exponential rate [4]. Thus, manual collection of relevant published exposure information on one or several chemicals is an extremely time consuming task. Furthermore, due to the broad variety of methods used for exposure estimations and the large amount of studied exposure routes and sources, manual searches in commonly used search engines e.g. Google Scholar (http://scholar.google.com) or PubMed (http://www.ncbi.nlm.nih.gov/PubMed) require a large number of search terms, and still articles will be missed due to unsuitable or lack of search terms.

Text mining (TM) is the process of acquiring refined information by analyzing correlations and statistical patterns in unstructured text. Over recent years, TM techniques have enabled large-scale information extraction and knowledge discovery [5], and have been successfully applied in real life tasks. For example, TM techniques have been applied in cancer research [6, 7] and cancer chemical risk assessment [8, 9], toxicogenomics [10], and drug effects/safety [11, 12].

In this paper, we describe the first steps towards developing a semantic text classifier for text mining human exposure information. A classifier for exposure data can be used to rapidly retrieve and categorize exposure data, map chemical-specific exposure information, identify research gaps and create new research ideas. We apply proven Natural Language Processing methodology and evaluate the performance using both intrinsic evaluation and case studies.

## 2. Methods

### 2.1. The exposure information corpus

#### 2.1.1. Taxonomy

An exposure taxonomy was developed by experts in biomonitoring and exposure sciences to categorize data into relevant classes. The taxonomy only regards human exposure data, thus animal and *in vitro* data are not included. The taxonomy includes a total of 32 nodes divided under two main branches of exposure information; *biomonitoring* and *exposure routes*. The first main branch categorises for *biomonitoring* data and is further divided into *exposure biomarkers* (i.e. chemicals or chemical metabolites measured in human tissues to assess exposure) and *effect biomarkers* (i.e. markers of effects caused by chemical exposures). The *exposure biomarker* branch is further structured based on the biological matrix (e.g. *blood*, *urine*, *hair/nail*) in which the biomarker has been measured. The *effect biomarker* branch is further structured based on the character of the marker (i.e. *molecule*, *gene*, *oxidative stress marker*, *other effect biomarker and physiological parameter*).

The second main branch contains data about the studied *exposure routes*, i.e. *oral intake*, *inhalation*, *dermal exposure* and *combined* exposure routes. These nodes are further structured into specific types of studied exposure. The information in the exposure routes branch includes e.g. mathematical models of exposure (dietary intake assessments, physiologically based pharmacokinetic (PBPK) modeling, etc.) and information obtained via questionnaires (consumption frequency of certain foods, use of consumer products, etc.). If chemicals were measured in an exposure media (e.g. food or water) without any exposure estimate on individual or population level, the abstract was regarded as irrelevant. However, if chemicals were measured in indoor air or dust in homes or workplaces without exposure estimates, the information was still considered relevant because the exposure levels regard an indoor environment specific for a group of residents or workers. See Table 1 for examples of information considered relevant for different nodes.

**Table 1:** Examples of information considered relevant for different nodes in the exposure taxonomy.

Node			Relevant information
**BIOMONITORING**			
	**Exposure biomarker**		
		*- Blood*, *urine*, *hair/nail*, *adipose tissue*, *mother’s milk*, *placenta*, *other tissue*	Measurements of exposure biomarkers (chemicals or metabolites) in corresponding human matrix (blood, urine, etc).
	**Effect biomarker**		
		*- Gene*, *molecule*, *oxidative stress marker*, *other effect biomarker*	Measurements of effect biomarkers in human matrices.
		*- Physiological parameter*	Measurements of physiological markers of effect, such as blood pressure, lung function, birth weight, etc.
**EXPOSURE ROUTES**			
	**Combined**		Intake calculations derived from biomonitoring data. Exposure modelling (e.g. PBPK) of multiple exposure routes simultaneously. Job exposure matrix.
	**Dermal exposure**		Tape strip samples, hand wipes, hand washing samples, dermal wipes, dermal exposure modelling.
	**Inhalation**		
		*- Outdoor air*	Data from ambient air monitoring stations used in exposure assessments or epidemiological studies.
		*- Indoor air*	Air in indoor microenvironments (homes, workplaces, schools, cars, etc). Environmental tobacco smoke. Inhalation from showers, cooking fuel, etc.
		*- Personal air*	Personal air monitoring, breathing zone measurements.
	**Oral intake**		
		*- Drinking water*	Exposure estimates from drinking water, bottled water, well water, etc.
		*- Dust*	Dust in indoor microenvironments (homes, workplaces, schools, cars, etc).
		*- Food*	Exposure estimates from food (e.g. intake assessments based on food concentration data and ingested amount of food, total diet studies, double portions, etc).
		*- Products*	Exposure estimates from toys, cosmetics, personal care products, dental fillings, drugs and vaccines, household pesticides, etc.
		*- Soil*	Exposure estimates from playground soil or residential garden soil, etc.

#### 2.1.2. Literature retrieval and annotated corpus

The development of the classifier requires a training data set consisting of a selection (i.e. corpus) of PubMed abstracts manually annotated according to the exposure taxonomy. The selection of the annotated text material included abstracts in scientific journals representing a variety of research fields, and published over a long time period. Seventeen well studied compounds or groups of compounds representing different exposure routes and sources were selected for the first annotation round. These compounds were bisphenol A, phthalates, polychlorinated biphenyls (PCB), polybrominated diphenyl ethers (PBDE), perfluorooctanesulfonic acid (PFOS), chlordane, cadmium, arsenic, mercury, acrylamide, triclosan, naphthalene, benzene, styrene, toluene, particulate matter 2.5 and chlorpyrifos.

First, abstracts published between 1993 and 2013 were selected using a search term including the 17 chemicals mentioned above and 24 journals within in the fields of epidemiology, toxicology, exposure and environmental sciences. Because the exposure taxonomy only concerns human exposure data and not *in vitro* and animal studies, only abstracts indexed with the Medical Subject Heading (MeSH) “humans” in PubMed were included in the annotated corpus. The search generated 5014 abstracts that were reviewed by an expert in exposure sciences and classified according to the taxonomy. The abstracts were only annotated if considered relevant for the taxonomy and consequently 2098 abstracts were annotated.

After the initial broad search and annotations, several sub-nodes contained an insufficient number of annotated abstracts. Thus, additional searches using targeted keywords were performed. Some of these additional search strings included other journals and chemicals than the ones used the initial searches. The targeted searches generated 2748 abstracts, out of which 1588 abstracts were relevant and annotated.

In total, the annotated text corpus included 3686 annotated abstracts. In addition, 4076 abstracts were reviewed but considered irrelevant and were not annotated. Table 2 shows the number of annotated abstracts for each node in the taxonomy.

**Table 2:** Number of annotated abstracts for each node in the taxonomy.

Node					# abstracts
**BIOMONITORING**					8
	**Exposure biomarker**				106
		Adipose tissue			88
		Blood			744
		Hair/nail			418
		Mother’s milk			177
		Other tissue			143
		Placenta			60
		Urine			784
	**Effect biomarker**				78
		Biomarker			27
			*Gene*		141
			*Molecule*		52
				*Lipid*	94
				*Other molecule*	168
				*Protein*	300
			*Other effect biomarker*		65
			*Oxidative stress marker*		62
		Physiological parameter			777
**EXPOSURE ROUTES**					168
	**Combined**				165
	**Dermal exposure**				153
	**Inhalation**				356
		Outdoor air			247
		Indoor air			254
		Personal air			174
	**Oral intake**				63
		Drinking water			424
		Dust			256
		Food			647
		Products			164
		Soil			131

The annotation was conducted by an expert with 5 years of experience in exposure assessment. The XUL-based annotation tool described in Guo et al., 2012 [13] was used with its menu items customized to our exposure information classification task. To investigate the accuracy of annotations, we performed inter-annotator agreement analysis where a second expert annotator was asked to annotate a subset of 200 abstracts. The annotation was compared against that of the annotator who annotated the whole corpus. Near excellent agreement was found between the two annotators with the average Cohen’s Kappa of 0.79 across the whole taxonomy.

### 2.2. Natural language processing

**Fig 2:**
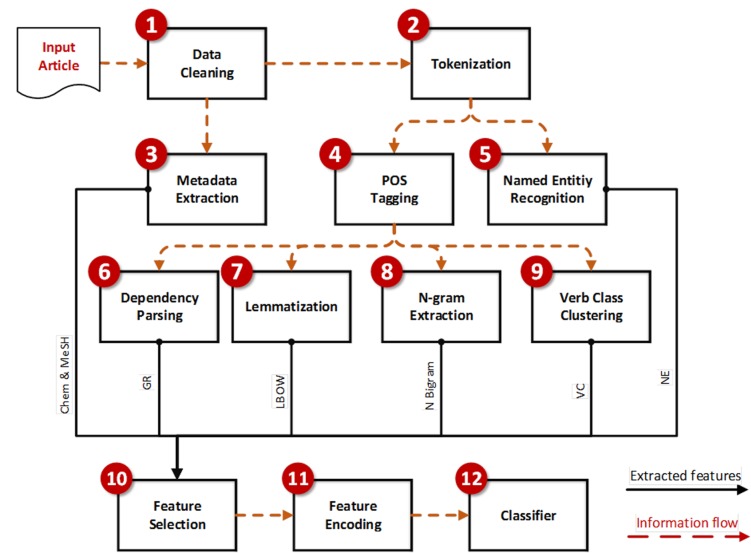
The NLP pipeline for automatic classification of document abstracts. Chem: Chemical lists, MeSH: Medical Subject Headings, GR: Grammatical Relations, LBOW: Lemmatized Bag of Words, N.Bigrams: Noun Bigrams, VC: Verb Clusters, NE: Named Entities.

In this section, we describe our Natural Language Processing (NLP) methodology used in the automatic classification. We applied proven NLP techniques and tools that have achieved state of the art results when trained with biomedical domain data. Fig 2 illustrates our handcrafted NLP pipeline.

The workflow of the NLP methodology is described below:

**1. Data Cleaning:** We first pre-process data retrieved from PubMed by extracting text appearing in the abstract, automatically cleaning the text from possible encoding errors.

**2. Tokenization:** We segment the text by words and then by sentences. We use BioTokenizer [14] to tokenize words and then Natural Language Tool Kit (NLTK) [15] for sentences. BioTokenizer achieves 96% Mean Average Precision, and is considered state of the art [14].

**3. Metadata Extraction:** Documents from PubMed are often accompanied with metadata that capture additional descriptive categorization information about the document. We extract two types of information: (i) the Medical Subject Headings (**MeSH**) that are used to tag biomedical documents with subject categories which are assigned by professional annotators [16], and (ii) a list of chemicals (**Chem**) that are referred to in the article.

**4. POS Tagging:** Part of Speech (POS) tagging is the process of labelling each word in the text with a lexical category label, such as *“noun”*, *“verb”*, and *“adjective”*. These labels are needed in subsequent stages in the pipeline. We use the C&C tagger [17, 18] which uses the Penn Treebank grammatical categories, and is trained on biomedical texts [19].

**5. Named Entity Recognition:** We identify and extract named entities that are typically discussed in the biomedical literature. We extract five named entity types that are common in biomedical text: DNA, RNA, proteins, cell line and type. We store in the feature a pair of the entity type and the associated words or phrases. We use the Named Entity Recognition (NER) tool ABNER [20], which is trained on the NLPBA and BioCreative corpora and achieves an F-score accuracy of 70.5% and 69.9% on these two corpora respectively [21]. By the end of this step, we extract Named Entities (**NE**) feature type.

**6. Dependency Parsing:** In this stage, we extract syntactic structures (trees) that encode grammatical dependency relations between words in the sentence. For example: direct object, non-clausal subject, and indirect object relations in parsed data taking into account their head and dependent words. We use the C&C parser to extract these grammatical relations. We trained the C&C Parser using available annotated biomedical annotated corpus [19]. We stored each arc in the tree (for all sentences in the given abstract) in a list. This list was used to generate the lexicalized GR features, which were essentially template “pattern” that we used to match during the feature encoding process. For example, a generated pattern could be (“subject <x>”, “affects”, “hemoglobin”) for a simple nsubj relationship. If an abstract has this pattern, 1 is assigned for the corresponding index for this feature.

**7. Lemmatization:** The goal of this stage of the pipeline is to produce Lemmatized Bag of Words **(LBOW)** features. Bag of words capture whether a word appears or not in a given abstract against all of the words that appear in the corpus. We lemmatize (stem) the text in order to reduce sparsity of the words occurring. We use the BioLemmatizer, which is trained on biomedical texts [22].

**8. N-gram Extraction:** We extract noun compound bigrams such as *“blood sample”*, or *“breast milk”*, as they can represent a concept in the text. We do not lemmatize the bigrams, as that could result in losing concept information, for example *“drinking water”*.

**9. Verb class Clustering:** We group semantically similar verb predicates together for example, verbs like *“stimulate”* and *“activate”* would be clustered together, compared to verbs like *“halt” and “slow”*. This allows us to generalize away from individual verbs and reduce data sparsity. We used the hierarchical classification of 399 verbs used in biomedicine by Sun and Korhonen [23]. We use three levels of abstraction by allocating 3 bits in our feature representation for each concrete class (1 bit for each level of the abstraction hierarchy). By the end of this stage, we extract the verb clusters (**VC**) feature type.

**10. Feature Selection:** Features that are too rare or too common in the annotated corpus are removed, so that only the most discriminating features are used by the classifiers. The thresholds are set for each node by a process of trial and error, typically a minimum threshold value of 5 occurrences are selected, while the maximum threshold varies greatly depending on the feature type; usually a value larger than 100. This improves both accuracy and reduces training time. This procedure is applied separately for each node in the taxonomy. Therefore, each classifier has a unique set of selected features. Table 3 details the number of features for each node after feature selection step. An additional feature ablation analysis (leaving one type of features out) allows a better understanding of which features are most informative for exposure information classification, as reported section 3.1.1.

**Table 3:** Feature selection. The number of features for each node in the taxonomy after the feature selection step.

Node					LBOW	GR	NE	VC	N Bigram	MeSH	Chem	Total
**BIOMONITORING**					4785	3544	352	128	1395	754	253	**11211**
	**Exposure biomarker **				4244	2965	312	127	1177	626	216	**9667**
		Adipose tissue			361	66	23	67	38	56	19	**630**
		Blood			2453	1300	141	122	521	355	111	**5003**
		Hair/nail			1390	532	47	109	266	186	34	**2564**
		Mother’s milk			668	197	43	91	85	86	35	**1205**
		Other tissue			612	106	14	92	66	82	21	**993**
		Placenta			307	52	13	80	32	37	13	**534**
		Urine			2310	1164	124	120	482	314	120	**4634**
	**Effect biomarker**				2889	1678	190	122	715	508	164	**6266**
		Biomarker			1743	733	146	116	363	313	129	**3543**
			*Gene*		637	141	39	96	79	96	25	**1113**
			*Molecule*		1461	555	121	115	271	248	103	**2874**
				*Lipid*	447	84	19	88	54	69	30	**791**
				*Other molecule*	724	181	37	97	99	106	40	**1284**
				*Protein*	1167	352	99	112	200	179	74	**2183**
			*Other effect biomarker *		357	44	16	73	35	38	11	**574**
			*Oxidative stress marker*		316	48	16	80	30	39	14	**543**
		Physiological parameter			2100	1018	78	121	434	347	77	**4175**
**EXPOSURE ROUTES**					4574	3356	248	130	1297	694	211	**10510**
	**Combined**				715	156	18	98	91	98	31	**1207**
	**Dermal exposure**				773	142	15	99	94	79	29	**1231**
	**Inhalation**				2607	1349	89	123	525	379	111	**5183**
		Outdoor air			1064	407	30	109	159	136	14	**1919**
		Indoor air			1308	369	35	107	191	178	58	**2246**
		Personal air			974	221	25	99	124	104	32	**1579**
	**Oral intake**				3216	2067	171	126	830	461	127	**6998**
		Drinking water			1338	501	44	117	250	191	45	**2486**
		Dust			1057	293	43	97	163	145	53	**1851**
		Food			1932	997	87	117	411	270	78	**3892**
		Products			803	193	18	97	117	99	29	**1356**
		Soil			652	124	5	84	86	80	24	**1055**
**Average:**					1328	624	67	100	271	193	61	**2645**

LBOW: Lemmatized Bag of Words, GR: Grammatical Relations, NE: Named Entities, VC: Verb Clusters, N.Bigrams: Noun Bigrams, MeSH: Medical Subject Headings, Chem: Chemical lists.

The corpus of annotated PubMed abstracts and the software for classification are available at: https://figshare.com/articles/Corpus_and_Software/4668229

**11. Feature Encoding:** The features are represented in a sparse binary format, with a value of 1 indicating that the given abstract contains this feature. The feature itself can be as simple as a bag of words, or as complex as carefully engineered grammatical relations (if there’s a certain dependency between specific word pairs), verb clusters (if there’s a verb instance belonging to a given verb class), NER (if there’s a specific word tagged with a specific named entity), etc.

**12. Classifier**: The binary features are then put into 32 classifiers (support vector machines with radial basis function kernels) that label each abstract with a binary label indicating its relevance for one of the 32 nodes in the taxonomy. Each of the classifiers are trained and executed independently in order to have mutually non-exclusive multi-label classification.

Each of the classifiers is trained with entire corpus data (unless we are evaluating the performance of the classifiers, where cross-validation is used). Abstracts that contain sentences annotated with its associated label, are counted as a positive example for the training, otherwise, it would be a negative example. We use the hypernym/hyponymy relationships in our taxonomy to determine whether an example should be labelled positively or negatively for a given node, i.e., we count sub-nodes labels as positive examples when we are classifying abstracts under their parent node.

### 2.3. Evaluation methodology

We evaluate the performance of our NLP methodology using an intrinsic evaluation (evaluating the NLP system’s performance with respect to our annotated data, i.e. gold data).

We use standard cross-validation setup, where the annotated gold dataset is split into four folds, the classifiers are trained on three of four folds and the system is tested on the remaining fold. We rotate the split until the entire annotated gold dataset is covered. We also apply another level of five-fold cross-validation for kernel hyper parameter tuning within the training split. The following standard measurements are used to ascertain the performance accuracy of our system:

$$precision=\frac{true\;postives}{true\;postives+false\;postives}$$

$$recall=\frac{true\;postives}{true\;postives+false\;negatives}$$

$$accuracy=\frac{true\;postives+true\;negatives}{total}$$

$$F\;score=2\frac{precision\times recall}{precision+recall}$$

## 3. Results

### 3.1. Intrinsic evaluation

Table 4 shows the results of the intrinsic evaluation using 3-fold cross validation.

**Table 4:** Results of intrinsic evaluation using 3-fold cross validation. All scores are percentages.

Node					Precision	Recall	Accuracy	F-score
***BIOMONITORING***					94.9	95.5	93.1	95.2
	**Exposure biomarker**				93.8	95.0	93.2	94.4
		Adipose tissue			93.9	87.5	99.6	90.6
		Blood			87.2	82.4	92.1	84.7
		Hair/nail			97.7	89.9	98.4	93.6
		Mother’s milk			91.6	86.4	99.0	89.0
		Other tissue			86.0	25.9	96.9	39.8
		Placenta			93.3	70.0	99.4	80.0
		Urine			95.7	91.9	97.1	93.8
	**Effect biomarker**				89.0	81.8	90.7	85.3
		Biomarker			89.4	69.2	94.2	78.0
			*Gene*		92.6	61.7	98.3	74.0
			*Molecule*		85.4	63.1	94.5	72.6
				*Lipid*	87.5	37.2	98.3	52.2
				*Other molecule*	80.0	42.9	96.9	55.8
				*Protein*	84.8	56.0	95.6	67.5
			*Other effect biomarker*		91.7	33.8	98.8	49.4
			*Oxidative stress marker*		82.1	51.6	99.0	63.4
		Physiological parameter			84.0	70.1	90.8	76.4
***EXPOSURE ROUTES***					89.0	92.3	87.4	90.6
	**Combined**				80.9	43.9	97.0	56.9
	**Dermal exposure**				83.9	72.6	98.0	77.8
	**Inhalation**				92.4	81.5	93.3	86.6
		Outdoor air			91.1	83.1	98.0	86.9
		Indoor air			78.6	30.4	92.3	43.8
		Personal air			90.5	74.8	97.9	81.9
	**Oral intake**				87.7	82.1	88.1	84.8
		Drinking water			83.4	81.3	95.7	82.3
		Dust			90.4	75.1	97.4	82.0
		Food			88.4	75.8	93.2	81.6
		Products			80.4	42.3	96.4	55.4
		Soil			78.5	62.5	97.7	69.6

**Fig 3:**
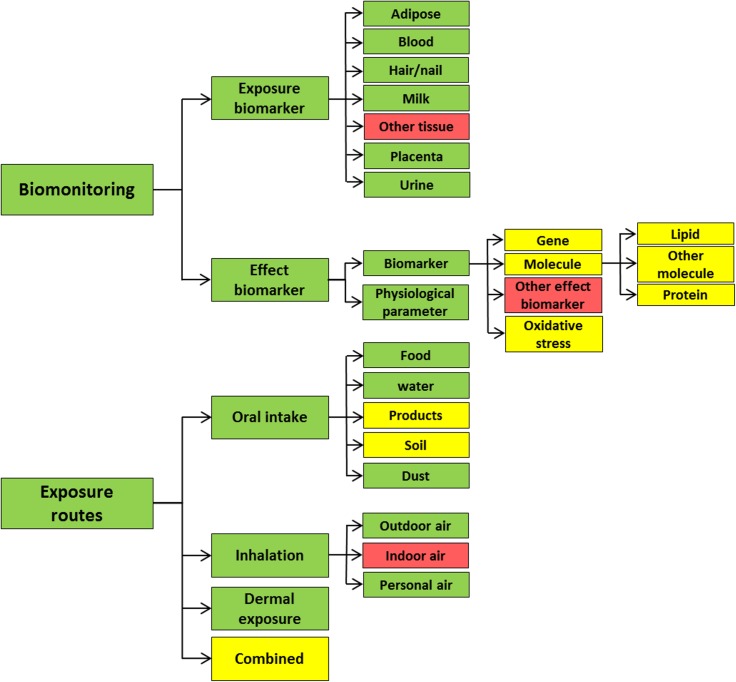
Results of the intrinsic evaluation. The color coding is based on F-scores (Green = >75%, yellow = 50–75%, red = <50%).

The intrinsic evaluation is also graphically presented in Fig 3 with color coding based on F-scores. The F-scores were generally high for the three top levels in the taxonomy. However, subdivision of effect biomarkers into distinct markers (i.e. gene, molecule and oxidative stress marker) generally resulted in lower F-scores. In addition, the nodes concerning *indoor air*, *other effect biomarker* and *other tissue* for exposure biomarker measurements performed poorly. Initially, the branches for exposure routes and exposure biomarkers were further classified into studied subpopulations (i.e. children, pregnant women and workers). However, due to the low number of abstracts relevant for these very specific sub-nodes, the resulting F-scores (range 11–78%) were not satisfactory and are therefore not reported in this paper.

#### 3.1.1. Feature analysis

In this section, we investigate the influence of each feature type on the classification accuracy. We conduct a leave-one-out feature analysis, where we repeat the experiment setup (described in the previous section), but with the removal of one of the seven feature types in order to ascertain its influence on the classification decision boundary for each node in the taxonomy. Table 5 summarizes the results of this analysis.

**Table 5:** Analysis of the influence of each feature type on the classification accuracy. The classification accuracy is described as the F-score for each node after removal of respective feature type. The column “all” describes the F-scores when all feature types are used. F-scores that decreased after removal of respective feature type are presented in bold script.

Node					All	LBOW	GR	NE	VC	N Bigram	MeSH	Chem
**BIOMONITORING**					95.2	**85.3**	**92.4**	**91.6**	**92.3**	**93.9**	**88.9**	**94.3**
	**Exposure biomarker**				94.4	**89.6**	**89.2**	**93.4**	**93.9**	**93.4**	**92.6**	**93.3**
		Adipose tissue			90.6	**80.6**	**84.0**	**87.2**	**79.5**	**83.7**	**79.1**	**84.3**
		Blood			84.7	**80.1**	**83.9**	**78.6**	**82.6**	85.2	**84.5**	**82.4**
		Hair/nail			93.6	**84.2**	**91.6**	**91.7**	95.3	**91.9**	**92.3**	97.0
		Mother’s milk			89.0	**72.0**	**85.4**	**88.9**	**83.1**	**78.6**	**76.8**	**76.2**
		Other tissue			39.8	**25.6**	**36.2**	**36.9**	**28.5**	**28.4**	**27.9**	**26.6**
		Placenta			80.0	**55.0**	**75.3**	**75.6**	**62.4**	**59.3**	**56.7**	**56.4**
		Urine			93.8	**88.8**	**90.1**	**93.3**	94.5	96.0	95.4	**92.0**
	**Effect biomarker**				85.3	**83.2**	**84.6**	**82.9**	**83.1**	**83.1**	**79.6**	**80.1**
		Biomarker			78.0	**72.3**	80.4	**73.9**	**76.9**	**73.2**	**70.0**	**71.7**
			*Gene*		74.0	**65.6**	**73.9**	**70.0**	**71.5**	**72.9**	**69.0**	**72.3**
			*Molecule*		72.6	**66.4**	76.7	**70.4**	**68.0**	**68.2**	**64.8**	**65.7**
				*Lipid*	52.2	**47.7**	**47.3**	**48.4**	53.2	53.4	**50.1**	53.3
				*Other molecule*	55.8	**46.6**	**53.6**	**55.1**	**45.4**	**47.5**	**49.4**	**46.0**
				*Protein*	67.5	**54.2**	**67.3**	**62.6**	**63.6**	**60.2**	**58.3**	**57.2**
			*Other effect biomarker*		49.4	51.5	**45.3**	**46.4**	54.8	54.7	54.7	53.3
			*Oxidative stress marker*		63.4	**62.7**	**62.7**	**59.2**	72.7	69.4	66.5	66.1
		Physiological parameter			76.4	**70.2**	**75.3**	80.4	**70.5**	**71.2**	**69.6**	**68.6**
**EXPOSURE ROUTES**					90.6	**89.2**	**85.6**	**84.5**	**86.4**	**89.7**	**85.0**	**88.9**
	**Combined**				56.9	**51.7**	**53.6**	57.2	**55.1**	**54.0**	**56.2**	**51.5**
	**Dermal exposure**				77.8	**71.2**	**71.9**	83.0	**74.9**	**74.8**	**73.6**	**71.4**
	**Inhalation**				86.6	**78.9**	**78.0**	88.5	**85.2**	**85.7**	**86.5**	**83.2**
		Outdoor air			86.9	**83.8**	**80.5**	**84.6**	87.3	**83.9**	**78.9**	**80.5**
		Indoor air			43.8	**43.6**	47.0	**43.2**	45.6	44.4	45.1	**43.6**
		Personal air			81.9	**77.8**	**74.8**	87.8	85.6	**81.5**	**76.8**	82.1
	**Oral intake**				84.8	**76.2**	**78.7**	**81.1**	87.2	**82.8**	**81.4**	**82.0**
		Drinking water			82.3	**73.8**	**76.1**	**80.9**	85.0	**80.9**	**80.2**	**78.7**
		Dust			82.0	**80.1**	**80.2**	**77.3**	82.4	**80.8**	**79.0**	**79.7**
		Food			81.6	**79.6**	**79.5**	**78.3**	**77.5**	**80.2**	**79.5**	**78.3**
		Products			55.4	**50.4**	**54.2**	**54.8**	56.0	**52.7**	**52.2**	**51.6**
		Soil			69.6	**58.0**	**68.8**	70.6	**62.2**	**65.2**	**61.0**	**64.8**
				**Average:**	75.5	**68.6**	**72.6**	**73.7**	**73.2**	**72.5**	**70.7**	**71.0**

LBOW: Lemmatized Bag of Words, GR: Grammatical Relations, NE: Named Entities, VC: Verb Clusters, N.Bigrams: Noun Bigrams, MeSH: Medical Subject Headings, Chem: Chemical lists.

On average, LBOW features have the most significant influence on the classification accuracy as it results in the largest drop (6.9%) on the averaged F-score. With the exception of only one category (*other effect biomarker*), all nodes benefit from the inclusion of LBOW features. On the other hand, the analysis shows that the removal of NE features marks the lowest drop (1.8%) in accuracy, with 6 out of the 32 categories showing an improvement in F-score. When considering only the count of nodes that show an improvement in F-score accuracy, VC features are the least beneficial overall, as 12 of the 32 categories improve in F-score when VC features are removed.

Overall, the analysis shows that all feature types benefit our classification methodology, as they all result in reduction of F-score when they are removed.

### 3.2. Case studies

The classifier was applied in a number of case studies demonstrating how the classifier can be used to assist researchers by facilitating information retrieval and classification, enabling data gap recognition and overviewing available scientific literature using chemical-specific publication profiles.

#### 3.2.1. Manual versus automatic search for data selection

One of the authors had previously performed an extensive manual search for articles that report levels of persistent organic pollutants (POPs) in human blood and breast milk. The manual search was performed while preparing for a report [24], in which a study was considered relevant for inclusion only if it fulfilled a set of criteria regarding sampling population, sampling year, methodical quality, etc. After applying these strict criteria, only a fraction of all articles reporting blood and milk measurements were included in the final report. However, in the initial stage it was crucial to find all potentially relevant articles for further manual evaluation, even if they did not meet the criteria for inclusion in the final report. Thus, the initial manual search was extremely time consuming.

This real life example was used to evaluate the applicability of the automatic classifier for the initial literature gathering and classification. Here, we compare the manual search with an automatic search performed by our classifier.

First, abstracts about the chemicals of concern published between 1 Jan 2000 and 1 July 2014 were retrieved from PubMed and classified by the automatic classifier. The comparison in Table 6 describes the recall of the classifier (i.e., was it able to retrieve most of the relevant abstracts), as shown in column “Automatic/Manual”. For measurements in blood, the automatic classifier found all articles that had been included in the report, with exception for one article. For measurements in milk, all except three articles were found by the automatic classifier. Table 6 also describes and the number of irrelevant abstracts that were successfully filtered out by the classifier (i.e., was it useful for reducing the amount of literature to be further manually reviewed by a researcher), as indicated by the difference between columns “Abstracts found with manual PubMed search” and “Abstracts automatically classified as …”. While there could be false positives in the abstracts automatically classified as relevant, meaning that the researcher still have to exclude some abstracts manually, the current case study does suggest that the automatic classifier offers a much better starting point for exposure assessment than traditional PubMed searches.

**Table 6:** Comparison between manual and automatic classification of articles describing measurements of nine chemicals/chemical groups in human blood and milk.

Compound	Abstracts found with manual PubMed search^1^	Measurements in blood		Measurements in mother’s milk	
		Abstracts automatically classified as blood^2^	Automatic/ Manual^3^	Abstracts automatically classified as milk^2^	Automatic/ Manual^3^
DDT/DDE	2050	604	28/28	137	5/5
α-, β- & γ-HCH	699	203	**14/15**	70	3/3
Mirex	86	46	2/2	11	0/0
PCB	3331	1152	30/30	232	10/10
PCDD/F	2886	480	5/5	162	5/5
PFOS	561	285	16/16	25	**6/8**
PBDE	905	251	23/23	130	**13/14**
Aldrin/ dieldrin	282	59	2/2	28	2/2
Endosulfan	257	37	2/2	13	1/1

^1^Number of abstracts found with a PubMed search using the chemical name as search term, applying time restriction 1 Jan 2000–1 July 2014 and only including abstracts indexed with the MeSH term “humans”.

^2^Number of abstracts automatically classified under respective node (blood or milk) regardless if they met the criteria for inclusion in the report.

^3^Number of manually selected abstracts that met the criteria for inclusion in the report (manual), and the number out of these manually selected abstracts that were found also among the abstracts automatically classified under each relevant node (automatic).

#### 3.2.2. Publication profiles of chemicals

Three chemicals (hexachlorobenzene, lead and 4-nonylphenol) with different properties, exposure routes and sources were selected to evaluate if publication profiles obtained from the automatic classification reflect expected exposure information profiles.

**Fig 4:**
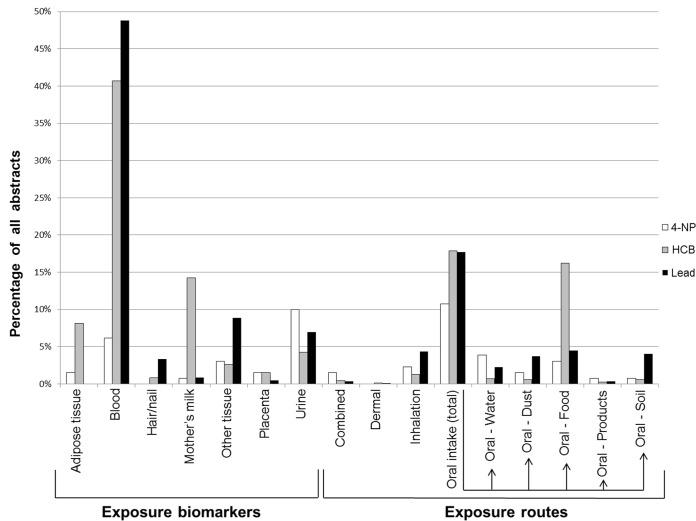
Publication profiles of exposure information about 4-NP, HCB and lead. The percentages of the total number of abstracts retrieved from PubMed and considered relevant for the full taxonomy are presented. The total number of abstracts was 130 for 4-NP, 722 for HCB and 7753 for lead.

*Hexachlorobenzene* (HCB) is a persistent organic pollutant which was used as a fungicide until the 1970s. Although it is now globally banned, HCB is still present in the environment and humans are exposed primarily via food [25, 26]. Fig 4 clearly illustrates that ingestion of food is the most studied exposure route, whereas other routes are poorly studied. HCB is a lipid-soluble compound predominantly measured in blood as well as in the body’s fat containing tissues, such as adipose tissues and mother’s milk, which is reflected by the publication profile.

*Lead* is a naturally occurring toxic metal used in e.g. mining and smelting activities and historically used in products such as paint and gasoline. Humans are mainly exposed to lead via ingestion of contaminated food or water and children can also be exposed via ingestion of dust and soil [27]. For occupationally exposed individuals, inhalation is an important exposure route. According to Fig 4, ingestion of food, dust, soil as well as inhalation are almost equally studied exposure routes. According to the publication profile, lead is predominantly measured in blood, followed by other tissues and urine, which is in accordance with current knowledge [28]. The high number of measurements in other tissues mostly considers lead in bones and teeth, which are established matrices for lead measurements.

A surprisingly high number of abstracts about lead and HCB were classified into the exposure biomarker sub-node urine. The reason for this is that other chemicals have been measured in urine in the same articles in which HCB or lead have been measured in blood. Likewise, articles about HCB and lead have been classified into the exposure route sub-node products because another chemical (mercury) has been measured in amalgam fillings (considered as a product) while exposure to HCB or lead has been assessed from other exposure sources than products in the same abstracts. The classifier has not misclassified the abstract in these cases, however it was not able to distinguish between different chemicals presented in the same abstract.

*4-nonylphenol* (4-NP) is an endocrine disrupting chemical that has been used as a surfactant in e.g. industrial and household cleaning products and in the production of e.g. paints and pesticides [29, 30]. Available human exposure information about 4-NP was found to be very scarce. Due to the low number of abstracts in each node for 4-NP, it is difficult to draw conclusions about the publication profile. However, Fig 4 shows that urine and blood are the most studied matrices for measuring exposure biomarkers, which is expected [31]. The low number of abstracts allowed manual evaluation of the classification precision of all abstracts about 4-NP included in Fig 4. With few exceptions, the abstracts were classified correctly (96% true positives, 4% false positives).

#### 3.2.3. Differences in exposure data within a group of chemicals

Phthalates are a group of industrial chemicals mainly used as plasticizers in PVC, but also in non-plastic products such as personal care products, paints and glues [32, 33]. Humans are exposed primarily via the diet, but also from dust, air and direct contact with consumer products [34–36].

This case study aims to map the distribution and amount of published exposure information available for six different phthalates; di(2-ethylhexyl) phthalate (DEHP), dibutyl phthalate (DBP), butylbenzyl phthalate (BBzP), diisobutyl phthalate (DiBP), diisononyl phthalate (DiNP) and diisodecyl phthalate (DiDP). Due to their toxic effects, the use of DEHP, DBP, BBzP and DiBP is prohibited within the EU since 2015, whereas DiNP and DiDP are still in use and now substitute the banned phthalates. In this case study, we investigate if the amount of available exposure data differs between the banned phthalates and the phthalates still in use.

**Fig 5:**
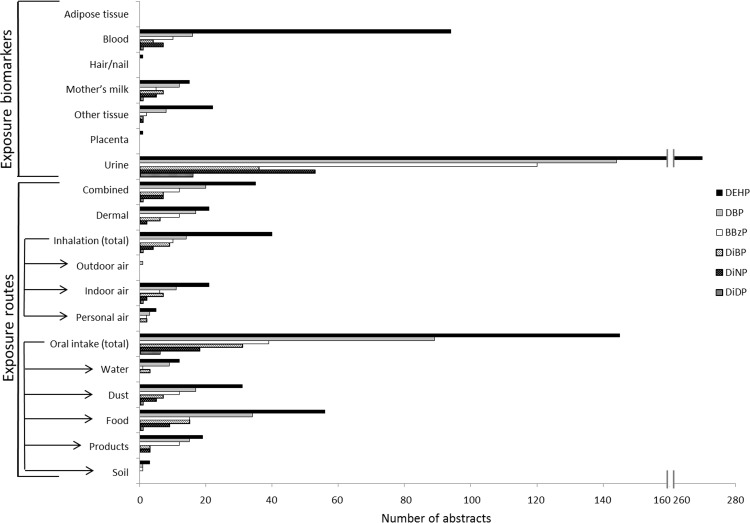
Publication profiles for exposure biomarkers and exposure routes for different phthalate esters.

**Fig 6:**
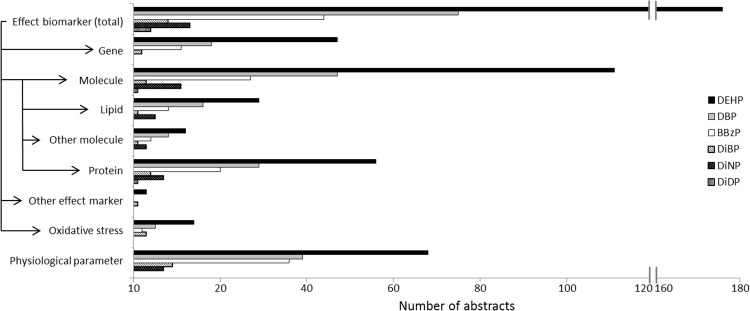
Publication profiles for effect biomarkers related to exposure to different phthalate esters.

All available abstracts in PubMed about these six phthalates were automatically classified to create publication distribution profiles. In Fig 5, publication profiles for exposure biomarker measurements and exposure routes are presented. The figure shows that the levels of phthalates in humans are measured predominantly in urine as phthalate metabolites, whereas measurements in blood are less common, which is expected. Measurements in adipose tissue, hair/nail and placenta are scarce or lacking for all phthalates. The publication profiles also reflect that we are mainly exposed to phthalates via food. Publication distribution profiles for effect biomarkers are presented in Fig 6.

This case study shows that the banned phthalates (DEHP, DBP, BBzP) are the most studied compounds, whereas there are considerable data gaps for the phthalates still in use (DiNP and DiDP). However, there are little available data also for the banned phthalate DiBP. The relatively larger amount of data for the banned compounds may be due to that these compounds have more established toxic properties and have historically been the most commonly used phthalates. However, DiNP and DiDP are among the most used phthalates in Europe today [33, 37], which is a reason to request more exposure data for these compounds. The overall pattern of phthalates may reflect general lack of exposure data for substituting compounds, whereas ample exposure data are available for compounds used for a long time. These findings raise concern that the research is not up-to-date with the current use of chemicals.

#### 3.2.4. Information retrieval

Automatic classification of abstract text can be shown to improve information retrieval compared to manual PubMed searches. The aim of this case study was to show whether using our classification system we can improve the recall of abstracts while at least keeping the same precision of results retrieved by PubMed search queries.

In this case study, we considered search queries that utilize our classification taxonomy. We selected the pollutant *lead* (CAS number 7439-92-1) as the chemical of interest and we then retrieved abstracts from PubMed that are relevant to a certain type of exposure, for example inhalation, i.e. the search query would typically be formulated as “*7439-92-1 AND inhalation*” which retrieves abstracts from PubMed that should in theory be both relevant to lead and inhalation. This particular search query yielded 149 results. However, when using our system in conjunction, i.e. by classifying all lead related documents according to the taxonomy, and then observe how many were classified under the *inhalation* node in the taxonomy, we identified 337 abstracts, which is more than double the number identified by PubMed. This large difference is due to the fact that our NLP pipeline considers far more factors than search term occurrences in the text.

In this case study, we show how this works by running this experiment on the five search queries shown in Table 7, along with automatic classification into the corresponding nodes in our taxonomy. Table 7 details the improvement in recall for the five queries when using our automatic classifier in comparison to the corresponding search query in PubMed.

**Table 7:** The number of abstracts retrieved by PubMed using a search query VS the number of abstracts classified into the corresponding node in our system.

PubMed search query	Node in the taxonomy	# of abstracts retrieved by PubMed^1^	# of abstracts classified by our system^1^
**7439-92-1 AND inhalation**	Exposure routes → Inhalation	149	337
**7439-92-1 AND (DNA OR gene) AND biomarker**	Biomonitoring → Effect biomarker → Biomarker → Gene	65	120
**7439-92-1 AND protein AND biomarker**	Biomonitoring → Effect biomarker → Biomarker → Molecule → Protein	149	357
**7439-92-1 AND blood AND biomarker**	Biomonitoring → Exposure biomarker → Blood	407	3784
**7439-92-1 AND (hair OR nail) AND biomarker**	Biomonitoring → Exposure biomarker → Hair/nail	24	257

^1^These numbers include both true and false positive abstracts.

We can see that our system was able to identify far more abstracts than what was retrieved by PubMed, in some cases by an order of magnitude. However, improving recall alone is not sufficient, as we also need to at least keep the same level of precision currently achieved by PubMed. In order to approximate this, the top 20 abstracts retrieved from each PubMed search query were reviewed to ascertain whether each abstract is relevant for the given query. From this relevance judgment, we can approximate the precision and therefore percentage of false positive abstracts from the PubMed search as well as our system’s classification. Table 8 details the precision performance according to the top 20 retrieved abstracts.

**Table 8:** Performance comparison according to top returned results sample. Manual evaluation of 20 abstracts retrieved from PubMed using a search query VS automatic classification into the corresponding node in our system.

PubMed search query	PubMed keyword search		Classification by our system		Sample size
	Precision	False Positive	Precision	False Positive	
7439-92-1 AND inhalation	50%	50%	100%	0%	20
7439-92-1 AND (DNA OR gene) AND biomarker	35%	65%	100%	0%	20
7439-92-1 AND protein AND biomarker	35%	65%	100%	0%	20
7439-92-1 AND blood AND biomarker	85%	15%	95%	5%	20
7439-92-1 AND (hair OR nail) AND biomarker	60%	40%	75%	20%	20

From the results in Table 8, we observe that our model’s performance is substantially more precise and has far fewer false positives than the keyword based search in PubMed. We believe that in the cases where search queries have correspondence to our taxonomy, we can improve both recall and precision by a substantial margin. This is due to the fact that our classification system considers far more factors when judging for relevance according to the exposure domain than PubMed keyword searches. Therefore, our specialised system can provide superior information retrieval for exposure science researchers.

## 4. Discussion

In this paper, we introduce a new application of text mining technology in the scenario of exposure research. We have evaluated the performance of the system and presented a number of case studies showing the usefulness of an automatic classifier for retrieving and classifying exposure information when e.g. preparing for reports, reviews or exposure assessments and for overviewing publication distribution profiles and identifying data gaps.

This project benefits from the collaboration between experts in exposure sciences, who have developed a relevant taxonomy and performed the annotations applying their knowledge in the field, and scientists in computational linguistics. Although text mining approaches have been developed and used for several biomedical fields [5], this is the first automatic classifier for chemical exposure information. We used traditional, but still top-performing feature extraction and machine learning algorithms for semantic classification of biomedical literature [38–40]. The SVM classifier used in our study has important advantages for text classification, being fairly robust to overfitting and easy to scale up to considerable feature dimensionalities [40]. Even the state-of-the-art document classification technology is built on top of standard classifiers such as a logistic classifier or SVM [41]. Recent advances in representation learning enable words, sentences, and documents to be represented by a dense vector which, on certain datasets, outperforms n-grams and alike obtained through traditional feature engineering. However, counterexamples also exist especially in similar classification tasks on biomedical literature data. For instance, as reported in [42], without the assistance of handcrafted features, the performance of the more fancy distributed representations of documents is less satisfactory, justifying the use of classical features.

The intrinsic evaluation of our automatic classifier showed good performance as high F-scores were generally achieved. We have demonstrated that our classifier successfully categorizes exposure data in a manner that greatly narrows down the amount of irrelevant information that would be retrieved by broad keyword based PubMed searches. We have also showed that our classifier is capable of finding more abstracts in specific sub-nodes and at the same time achieve higher accuracy compared to if the corresponding PubMed search strings were used. Consequently, the information that would be lost using specified PubMed searches is found by our classifier and the relevance of the retrieved information is higher.

The problem-based case studies showed that the classifier successfully can be used by researchers as a time-saving complement to manual information search. We also showed that the classifier creates chemical-specific publication profiles that reflect what we know about well-studied chemicals, which further confirms the relevance of the obtained results. As we show in our case studies, such profiles can be used to overview exposure data when comparing different chemicals or compounds within a chemical group and to detect data gaps.

In this paper, we present the first automatic classifier for exposure data. To the best of our knowledge, the only existing database for annotated exposure information is the Comparative Toxicogenomics Database (CTD; http://ctdbase.org), in which exposure information is one out of several modules [43]. However, in contrast to our classifier, the CTD rely on manual curation of all articles in the database. The curation for the CTD is structured according to an Exposure Science Ontology (ExO) [44], which was not considered as a suitable structure for our exposure taxonomy.

The taxonomy presented in this paper has great potential to be extended to include additional relevant branches for exposure information. For example, measurements of environmental media, such as biota and ambient air may provide important complementary information, especially when other exposure data is scarce. The taxonomy could also be extended to include classification of health outcomes (i.e. disorders and/or diseases) of chemical exposures studied in e.g. epidemiological studies.

In our taxonomy and in the annotation process we exclusively considered human exposure data. In related research areas (such as toxicology), also animal and *in vitro* data are highly relevant for the chemical risk assessment. In future continuation of the project, animal and *in vitro* data could be included in the annotation and machine learning processes aiming to better overview the current knowledge of e.g. effect biomarkers.

In human exposure assessments, data from the general adult population is not necessarily transferrable to subpopulations, such as children or pregnant women, due to differences in behavioral patterns, physiology and susceptibility. Chemical exposure measurements or calculations representative for specific subpopulations are therefore important to accurately assess exposure in the total population and to identify highly exposed groups in the population [45]. Therefore, the taxonomy was initially developed to differentiate between studies performed in children, pregnant women and workers. The classifiers achieved F-scores of 11–78 percent for these subpopulation specific nodes under the exposure biomarker and exposure route branches (data not shown). These moderate scores may be due to the relatively few abstracts available for annotation for these sub-nodes. This highlights the need for more exposure studies performed in susceptible subgroups.

The evaluation of the classifier revealed some challenges which should be addressed in further optimization of the system. Here, we present three challenges that should be overcome. 1) The nodes *other tissue* for exposure biomarker measurements, *other effect biomarkers* and *indoor air* performed poorly, probably due to the diversity of data in these nodes. Using our gained knowledge about the characteristics of this diverse information, each node can be divided into more specific nodes, which provide more relevant classification and enhance the F-scores. 2) The classifier is unable to distinguish between different chemicals that are measured in different tissues but presented in the same article. This is not a classification error *per se* since these abstracts are classified correctly based on the available information, however chemical-specific publication profiles may show an incorrect pattern if the misclassification occurs systematically. Addressing this problem will require further analysis of the available annotations for textual cues that might help the classifier to make the distinction. 3) Aiming to exclude animal and *in vitro* studies when retrieving abstracts from PubMed, only abstracts indexed with the MeSH term “humans” were used. However, some animal and *in vitro* studies were also labeled with this MeSH term and were therefore retrieved and included in the automatic classification. In these cases, our classifier classified the information correctly, but the interpretation of the results as exclusively human data may be misleading. To overcome this problem, we can’t change the manner in which abstracts are indexed by PubMed. However, by extending the taxonomy to include animal and *in vitro* studies, we would overcome these biases.

In conclusion, we have introduced and evaluated an automatic classifier for human exposure data that constitutes the first step towards developing a text mining tool that can support practical tasks such as the exposure assessment in the chemical risk assessment process. The promising results reported in this paper and identification of strengths, challenges and possible future improvements of the classifier can be used to further optimize the approach in future work.
